# Diagnosis of Subcortical Ischemic Vascular Cognitive Impairment With No Dementia Using Radiomics of Cerebral Cortex and Subcortical Nuclei in High-Resolution T1-Weighted MR Imaging

**DOI:** 10.3389/fonc.2022.852726

**Published:** 2022-04-08

**Authors:** Bo Liu, Shan Meng, Jie Cheng, Yan Zeng, Daiquan Zhou, Xiaojuan Deng, Lianqin Kuang, Xiaojia Wu, Lin Tang, Haolin Wang, Huan Liu, Chen Liu, Chuanming Li

**Affiliations:** ^1^ Department of Radiology, Second Affiliated Hospital of Chongqing Medical University, Chongqing, China; ^2^ Department of Radiology, Third Affiliated Hospital of Chongqing Medical University, Chongqing, China; ^3^ Department of Radiology, The Second People’s Hospital of Jiulongpo District, Chongqing, China; ^4^ Department of Ultrasound, Chongqing Maternal and Child Health Hospital, Chongqing, China; ^5^ Medical Data Science Academy, Chongqing Medical University, Chongqing, China; ^6^ Department of Data Analysis, GE Healthcare, Shanghai, China; ^7^ Department of Radiology, The First Affiliated Hospital of Army Medical University, Chongqing, China

**Keywords:** subcortical ischemic vascular cognitive impairment with no dementia, diagnosis, radiomics, high-resolution T1-weighted imaging, machine learning

## Abstract

**Purpose:**

To investigate whether the combination of radiomics derived from brain high-resolution T1-weighted imaging and automatic machine learning could diagnose subcortical ischemic vascular cognitive impairment with no dementia (SIVCIND) accurately.

**Methods:**

A total of 116 right-handed participants involving 40 SIVCIND patients and 76 gender-, age-, and educational experience-matched normal controls (NM) were recruited. A total of 7,106 quantitative features from the bilateral thalamus, hippocampus, globus pallidus, amygdala, nucleus accumbens, putamen, caudate nucleus, and 148 areas of the cerebral cortex were automatically calculated from each subject. Six methods including least absolute shrinkage and selection operator (LASSO) were utilized to lessen the redundancy of features. Three supervised machine learning approaches of logistic regression (LR), random forest (RF), and support vector machine (SVM) employing 5-fold cross-validation were used to train and establish diagnosis models, and 10 times 10-fold cross-validation was used to evaluate the generalization performance of each model. Correlation analysis was performed between the optimal features and the neuropsychological scores of the SIVCIND patients.

**Results:**

Thirteen features from the right amygdala, right hippocampus, left caudate nucleus, left putamen, left thalamus, and bilateral nucleus accumbens were included in the optimal subset. Among all the three models, the RF produced the highest diagnostic performance with an area under the receiver operator characteristic curve (AUC) of 0.990 and an accuracy of 0.948. According to the correlation analysis, the radiomics features of the right amygdala, left caudate nucleus, left putamen, and left thalamus were found to be significantly correlated with the neuropsychological scores of the SIVCIND patients.

**Conclusions:**

The combination of radiomics derived from brain high-resolution T1-weighted imaging and machine learning could diagnose SIVCIND accurately and automatically. The optimal radiomics features are mostly located in the right amygdala, left caudate nucleus, left putamen, and left thalamus, which might be new biomarkers of SIVCIND.

## Introduction

Dementia is a syndrome that involves severe loss of cognitive abilities as a result of disease or injury. It is a serious threat to the elderly and a heavy burden for society. Globally, the prevalence of dementia in the elderly over 65 is estimated to be as high as 7%; besides, it can reach 8%–10% in developed countries because of longer life spans (1). It often leads to the decline of intelligence, memory, orientation, computing, and comprehension, which can be accompanied by decreased language ability and emotional and personality changes (2). Vascular dementia (VaD) is the second most common type of dementia. Subcortical ischemic vascular cognitive impairment with no dementia (SIVCIND) is considered to be a prodromal stage of subcortical ischemic VaD (SIVD), which is an important subtype of VaD (3). Early diagnosis of SIVCIND has important clinical value because timely treatment can greatly prevent disease development and improve the prognosis (4).

Until now, the clinical diagnosis of SIVCIND is mainly based on neuropsychological scale testing, electrophysiological examination, and the evidence of subcortical cerebrovascular disease from clinical data or medical imaging. However, a formal neuropsychological evaluation is often time-consuming and lacks objectivity (5), while electrophysiological examination usually lacks specificity (6), which limits their clinical use greatly. In recent years, neuroimaging utilizing structural or functional methods has been regarded as a promising tool. Compared to the normal control group, the subcortical VaD group exhibited cortical atrophies in the frontal, occipital, and temporal areas and low integrity in the genu and splenium parts of the corpus callosum (7). Li et al. (8) also found that SIVCIND patients showed significant cerebral gray matter volume reduction in the insula, superior temporal gyrus, hippocampus, and parahippocampal gyrus, which have a closed correlation with language dysfunction, memory loss, and attention deficits. However, most of these studies only analyzed the changes in the macro structure of the brain, ignoring the micro changes in the subtle structure invisible to the naked eye (7–9).

Radiomics can extract high-dimensional image features from medical imaging (CT, MRI, positron emission tomography (PET), etc.) and analyze these features using high-throughput quantitative methods (10). Machine learning uses statistical techniques to grant computer systems the capability to “learn” from data to promote performance on an exact task without being explicitly programmed (11). The combination of radiomics and machine learning could help doctors diagnose diseases, evaluate prognosis, and even explore the correlation between images and genes. Recently, using this method, Betrouni et al. (12) proved that the texture features of the hippocampus and entorhinal cortex can help diagnose early cognitive impairment after stroke. The radiomics features of skewness and entropy in the hippocampus, thalamus, and amygdala were also found to be significantly different between the Parkinson’s group and healthy controls (13). Tozer et al. (14) reported that the texture parameters of white matter lesions could help diagnose cognitive impairment in cerebral small vessel disease and correlated with the global function and executive function significantly. However, until now, there is no study of the automatic diagnosis of SIVCIND by using radiomics and machine learning. In this study, we tried to use high-resolution T1-weighted imaging (T1WI) images to analyze the whole cerebral cortex and subcortical nuclei changes by radiomics to find characteristic biological features of SIVCIND and combine machine learning to establish diagnostic models to promote its early diagnosis.

## Materials and Methods

### Patient Cohort

This retrospective study was approved by the medical ethics committee of our hospital. All participants provided informed consent. A total of 116 right-handed participants involving 40 SIVCIND patients and 76 normal controls were recruited. Clinical performance was evaluated with the following neuropsychological tests: Mini-Mental State Examination (MMSE), Clinical Dementia Rating (CDR), Global Deterioration Scale (GDS), Activities of Daily Living Scale (ADL), Montreal Cognitive Assessment (MoCA), and Hachinski ischemic score (HIS) (15–17). According to the criterion proposed by Galluzzi et al. (18), only the SIVCIND patients who had a subcortical white matter hyperintensity (WMH) on T2-weighted imaging and had at least two lacunar infarcts were enrolled in this study. The SIVCIND patients were diagnosed in light of the following criteria (19): 1) subjective cognitive impairment reported by participants or their caregivers 2) insufficient cognitive deficits to reach the fifth revision of Diagnostic and Statistical Manual of Mental Disorders (DSM-5) for dementia; and 3) neuropsychological examination containing HIS judgment (HIS ≥ 7). Exclusion criteria included organic lesions of the brain (i.e., traumatic brain injury, acute phase of encephalorrhagia, epilepsy, encephalitis, encephaloma, and Parkinson’s disease), somatic disease (i.e., severe organ dysfunction syndrome, malnutrition, infection, anemia, and hypothyroidism), and mental illness that may influence cognitive abilities such as schizophrenia. Seventy-six gender-, age-, and educational experience-matched healthy volunteers were recruited as the normal controls, and all of them had no nervous system illness. None had current or a history of psychiatric diseases or risk factors of blood vessels that could lead to cognitive impairment. None of them had brain neoplasms, brain trauma, systemic illness, or MRI contraindications.

### MRI Acquisition

All of the MR images were obtained on a 3.0-T scanner that was equipped with eight-channel phased-array head coils (Magnetom Trio; Siemens Medical Systems, Erlangen, Germany). The head motion minimization was controlled by foam padding. The high-resolution T1WI images were obtained by magnetization-prepared rapid gradient-echo (MP-RAGE) sequence (repetition time/echo time/inversion time (TR/TE/TI) = 1,900/2.52/900 ms, thickness = 1.0 mm, no gap, 176 slices, matrix = 256 × 256, voxel size = 1 mm × 1 mm × 1 mm, flip angle = 9°). The conventional MRI sequences were as follows: T1WI images (TR/TE = 200/2.78 ms, thickness = 4.0 mm, 25 slices, matrix = 384 × 384, voxel size = 0.7 mm × 0.6 mm × 5 mm, flip angle = 70°) and fluid-attenuated inversion recovery images (TR/TE/TI = 9,000/93/2,500 ms, thickness = 4.0 mm, 25 slices, matrix = 256 × 256, voxel size = 0.9 mm × 0.9 mm × 4 mm, flip angle = 130°).

### Data Processing and Radiomics Feature Extraction

The subcortical brain region segmentation was performed by the Brainnetome fMRI Toolkit^1^ and Statistical parametric mapping 12 software (SPM12, Wellcome Department of Cognitive Neurology, UCL, London, UK). First, the high-resolution T1WI images of each subject were converted to Neuroimaging Informatics Technology Initiative (NIFTI) format. Then, all data were normalized to the Montreal Neurological Institute (MNI) standard T1 template (standard space: 181 × 217 × 181, resolution: 1 mm × 1 mm × 1 mm). Meanwhile, the Brainnetome Atlas were resliced to the standard MNI space with a resolution of 1 mm × 1 mm × 1 mm. Fourteen gray matter nuclei were further extracted as masks including the bilateral thalamus, hippocampus, globus pallidus, amygdala, nucleus accumbens, putamen, and caudate nucleus. Finally, the volumes of interest (VOIs) were gathered by point multiplication of the normalized T1 images and these masks for each subject. Four hundred twenty-three radiomics features were quantitatively excavated from each gray matter nucleus by the In-house MATLAB script (20, 21). A total of 5,922 (423 × 14) radiomics features were acquired. These features could be grouped into three categories. Category 1: first-order statistics features, which quantitatively described the distribution of voxel intensity within the MR images. Category 2: textural features, which were calculated from the gray-level run-length matrix (GLRLM) and the gray-level co-occurrence matrix (GLCM) representing the heterogeneity of regions. Category 3: higher-order statistics features, which were derived from wavelet transformation of the first-order statistics features and texture features in eight directions (HLL, LLL, HLH, HHL, LLH, LHH, LHL, and HHH).

The feature extraction of the brain cortex was performed by FreeSurfer (v6.0.0) software^2^. First, the high-resolution T1WI images of each subject were converted to NIFTI data. The preprocessing included the following steps: correction of motion, stripping of the skull, transformation of coordinate, segmentation of gray–white matter, reconstruction of cortical surface models, labeling of a region, registration, and statistical analysis (22). Then the entire cortex was separated by the Destrieux atlas (23). Finally, eight brain cortex features from each brain region were obtained involving gray matter volume, surface area, SD of thickness, average thickness, folding index, intrinsic curvature index, integrated rectified mean curvature, and integrated rectified Gaussian curvature. A total of 1,184 (8 × 148) features were acquired.

### Feature Dimension Reduction, Model Construction, and Evaluation

**Figure 1:**
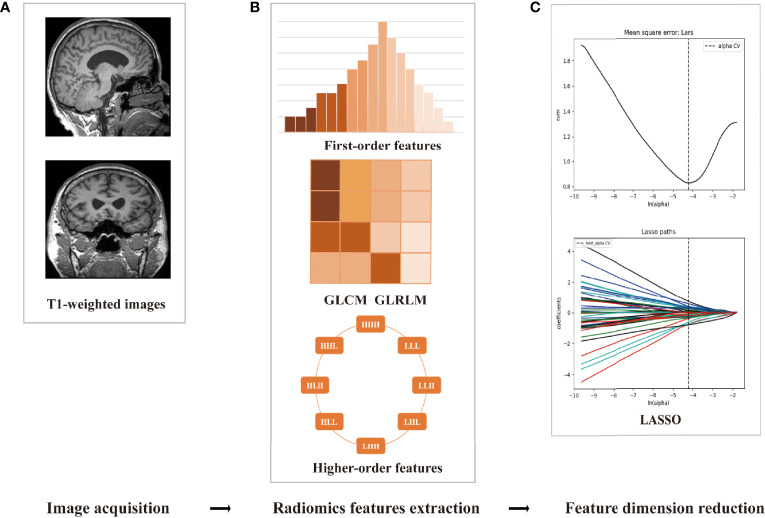
The framework of the radiomics workflow. **(A)** Data preprocessing and cortex/subcortical brain region segmentation. **(B)** Three categories of radiomics feature extraction. **(C)** Employing the least absolute shrinkage and selection operator (LASSO) algorithm to reduce the redundancy feature.

The processes of radiomics feature reduction and diagnosis model construction were performed with R software (24). First, the abnormal values were replaced by the median, and then standardization was applied to get all data on the same scale (25). The feature reduction included the following steps: univariate logistic regression (LR), Spearman’s correlation, general univariate analysis, gradient boosting decision tree (GBDT), least absolute shrinkage and selection operator (LASSO), and variance analysis (22). Then, three machine learning models of LR, random forest (RF), and support vector machine (SVM) were constructed. These machine learning models and feature selection methods were selected because of their practicability and experimental efficiency (11). Each model was trained and validated independently employing the 5-fold cross-validation, and 10 times 10-fold cross-validation was used to evaluate the generalization performance (11). The accuracy, specificity, sensitivity, and area under the receiver operator characteristic (ROC) curve (AUC) were employed to assess the diagnosis capability. The framework of the radiomics workflow is summarized in **Figure 1**.

### Correlation Analysis

Correlation analysis was performed between the optimal features and the MoCA, MMSE, and ADL scores of the SIVCIND patients using Pearson’s correlation test (for normally distributed features) or Spearman’s rank correlation analysis (for abnormally distributed features), respectively. A two-tailed *p* < 0.05 was regarded as statistically significant. SPSS (version 21.0) was used for correlation tests.

## Results

### Patient Cohort Characteristics

The SIVCIND patients showed significantly lower MoCA and MMSE scores than the normal controls (*p* < 0.001). There was no significant difference between the SIVCIND cohort and normal control cohort in gender (*p* = 0.589), age (*p* = 0.696), and educational experience (*p* = 0.773) (**Table 1**).

**Table 1:** Clinical characteristics and demographics of the SIVCIND and normal control subjects.

	NM (n = 76)	SIVCIND (n = 40)	*t-*Value	*p*-Value
Gender (male/female)	34/42	20/20	–	0.589^a^
Age (years)	62.9 ± 7.7(42–83)	63.6 ± 9.4(47–83)	−0.368	>0.05^b^
Education (years)	9.4 ± 4.0(0–17)	9.1 ± 4.3(0–17)	0.289	>0.05^b^
MoCA	27.1 ± 2.1(18–30)	18.6 ± 5.0 (6–26)	−10.124	<0.001^b^
MMSE	28.1 ± 1.5(23–30)	24.6 ± 3.0 (8–30)	−7.930	<0.001^b^
ADL	–	26.8 ± 10.4 (20–60)	–	–

Data are expressed as mean ± SD (range from min–max).

MoCA, Montreal Cognitive Assessment; MMSE, Mini-Mental State Examination; ADL, Activities of Daily Living Scale; NM, normal controls; SIVCIND, subcortical ischemic vascular cognitive impairment with no dementia.

a The p-value was acquired by Pearson’s chi-squared test.

b The p-value was acquired by two-sample t-test.

### Feature Dimension Reduction and Optimal Subset Selection

During the feature extraction, a total of 7,106 (5,922 + 1,184) features were acquired. A total of 5,922 features were from subcortical brain regions, and 1,184 features were from the brain cortex. First, 925 features were selected by employing the univariate LR. Then, with Spearman’s correlation, 413 features were selected. After general univariate analysis, 357 features were retained. Next, GBDT was employed, and 57 features remained. Then, 27 features were chosen through the LASSO method. Finally, 13 features were selected as the optimal subset with variance analysis. All these features are illustrated in **Table 2**.

**Table 2:** The radiomics features in the optimal subset.

Location	Category	Feature
Left putamen	First-order statistics features	CS
Left thalamus	First-order statistics features	Maximum
Right amygdala	Higher-order statistics features	Entropy-LHL
Right amygdala	Higher-order statistics features	HGLRE-LHL
Left caudate nucleus	Higher-order statistics features	SRE-HLH
Right hippocampus	Higher-order statistics features	LGLRE-LLH
Left nucleus accumbens	Higher-order statistics features	IMC2-LLL
Left nucleus accumbens	Higher-order statistics features	CS-HLH
Left nucleus accumbens	Higher-order statistics features	LRLGLE-LLH
Right nucleus accumbens	Higher-order statistics features	Correlation-HHH
Left putamen	Higher-order statistics features	Contrast-HHL
Left putamen	Higher-order statistics features	SRE-LLH
Left thalamus	Higher-order statistics features	SRE-HHH

Higher-order statistics features were derived from wavelet transformation including the first-order statistics features and texture features in eight directions (HLL, LLL, HLH, HHL, LLH, LHH, LHL, and HHH).

SRE, short run emphasis; LGLRE, low gray-level run emphasis; HGLRE, high gray-level run emphasis; CS, cluster shade; IMC2, informational measure of correlation 2; LRLGLE, long run low gray-level emphasis.

**Figure 2:**
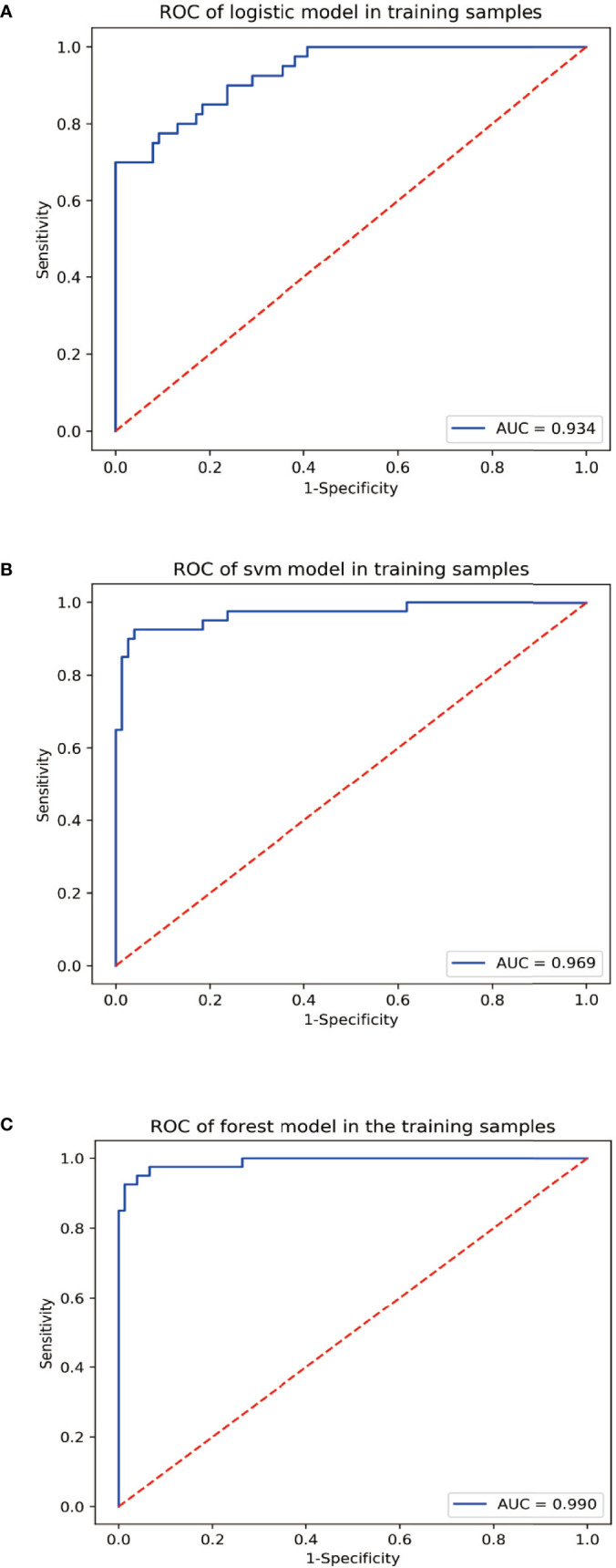
The receiver operator characteristic (ROC) curves of the radiomics models for discriminating the normal controls (NM) and subcortical ischemic vascular cognitive impairment with no dementia (SIVCIND) subjects. **(A)** ROC curve of logistic regression (LR) (area under the ROC curve (AUC) = 0.934). **(B)** ROC curve of support vector machine (SVM) (AUC = 0.969). **(C)** ROC curve of random forest (RF) (AUC = 0.990).

**Figure 3:**
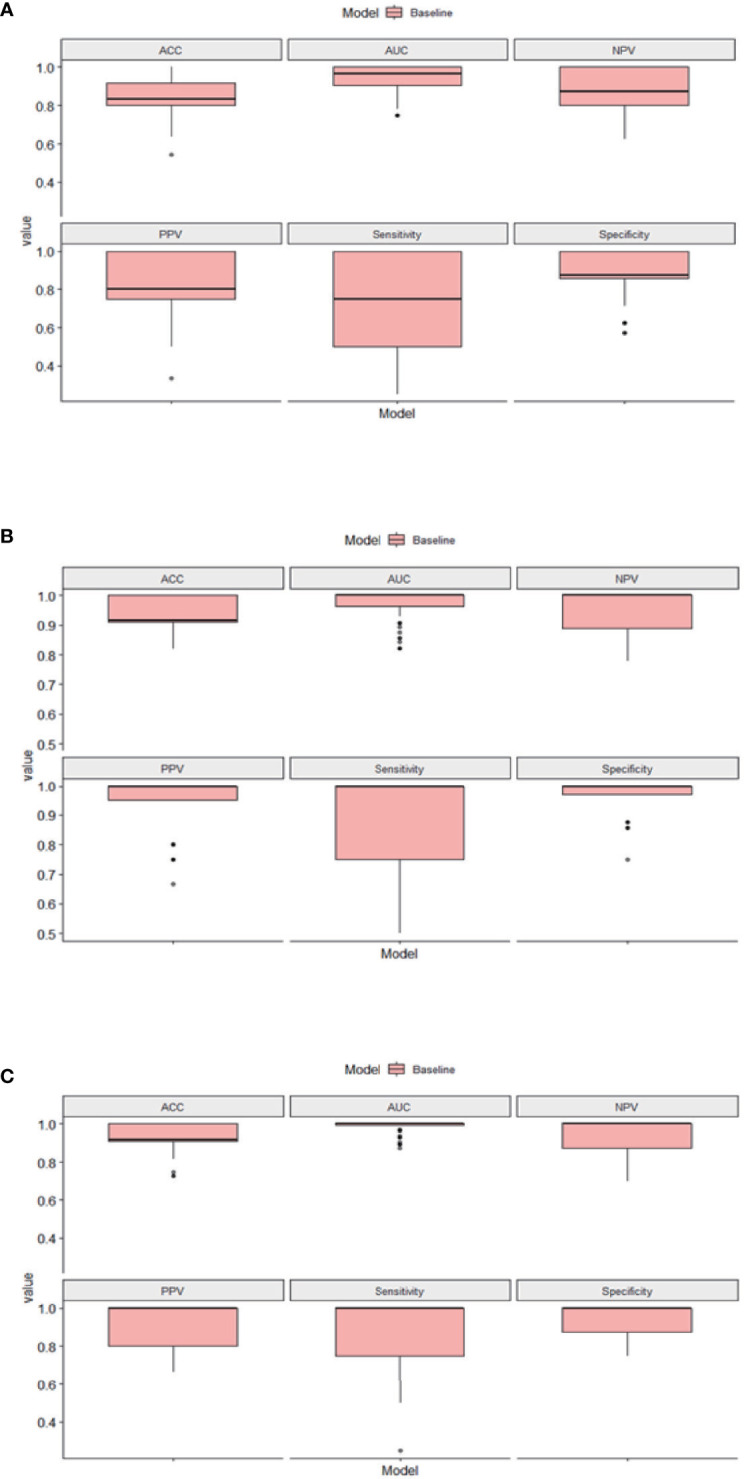
The results of the 10 times 10-fold cross-validation of our models. **(A)** Box plots of logistic regression (LR). **(B)** Box plots of support vector machine (SVM). **(C)** Box plots of random forest (RF). ACC, accuracy; AUC, area under the curve; NPV, negative predictive value; PPV, positive predictive value.

For the diagnosis of SIVCIND, the RF model, LR model, and SVM model showed an AUC of 0.990, 0.934, and 0.969; accuracy of 0.948, 0.888, and 0.888; sensitivity of 0.875, 0.675, and 0.700; and specificity of 0.987, 1.00, and 0.987, respectively. Among all the three models, the RF model yielded the best diagnosis performance (**Figure 2**). All of the models have robust generalization performance, and the results are expressed by box plots (**Figure 3**).

### Correlation Analysis

The High Gray Level Run Emphasis (HGLRE)-LHL of the right amygdala, Short Run Emphasis (SRE)-HLH of the left caudate nucleus and SRE-HHH of the left thalamus were found significantly correlated (p<0.05) with the MoCA scores of SIVCIND patients. The Cluster Shade (CS) of the left putamen and SRE-HLH of the left caudate nucleus were found significantly correlated (p<0.05) with the MMSE scores of SIVCIND patients. The SRE-HLH of the left caudate nucleus was found significantly correlated (p<0.05) with the ADL scores of SIVCIND patients. The detail results of the correlation test are shown in **Table 3**.

**Table 3:** Correlation tests between radiomics features of optimal subset and MoCA, MMSE, and ADL cores.

Item/location	Feature	Correlation coefficient	*p*-Value
MoCA			
Left putamen	CS	0.182	0.275
Left thalamus	Maximum	−0.111	0.508
Right amygdala	Entropy-LHL	0.256	0.121
Right amygdala^a^	HGLRE-LHL	0.425	0.008
Left caudate nucleus^a^	SRE-HLH	0.472	0.003
Right hippocampus	LGLRE-LLH	−0.089	0.597
Left nucleus accumbens	IMC2-LLL	−0.293	0.074
Left nucleus accumbens	CS-HLH	0.047	0.777
Left nucleus accumbens	LRLGLE-LLH	−0.052	0.756
Right nucleus accumbens	Correlation-HHH	0.153	0.358
Left putamen	Contrast-HHL	0.079	0.636
Left putamen	SRE-LLH	0.105	0.532
Left thalamus^a^	SRE-HHH	0.429	0.007
MMSE			
Left putamen^a^	CS	0.346	0.049
Left thalamus	Maximum	0.112	0.535
Right amygdala	Entropy-LHL	0.071	0.696
Right amygdala	HGLRE-LHL	0.072	0.689
Left caudate nucleus^a^	SRE-HLH	0.382	0.028
Right hippocampus	LGLRE-LLH	0.264	0.137
Left nucleus accumbens	IMC2-LLL	−0.033	0.855
Left nucleus accumbens	CS-HLH	−0.122	0.499
Left nucleus accumbens	LRLGLE-LLH	0.122	0.498
Right nucleus accumbens	Correlation-HHH	0.037	0.836
Left putamen	Contrast-HHL	−0.029	0.872
Left putamen	SRE-LLH	0.185	0.301
Left thalamus	SRE-HHH	0.254	0.154
ADL			
Left putamen	CS	−0.019	0.919
Left thalamus	Maximum	0.099	0.591
Right amygdala	Entropy-LHL	−0.059	0.747
Right amygdala	HGLRE-LHL	0.209	0.251
Left caudate nucleus^a^	SRE-HLH	−0.435	0.013
Right hippocampus	LGLRE-LLH	−0.148	0.418
Left nucleus accumbens	IMC2-LLL	−0.189	0.302
Left nucleus accumbens	CS-HLH	0.022	0.905
Left nucleus accumbens	LRLGLE-LLH	−0.213	0.242
Right nucleus accumbens	Correlation-HHH	−0.049	0.789
Left putamen	Contrast-HHL	−0.183	0.314
Left putamen	SRE-LLH	−0.031	0.865
Left thalamus	SRE-HHH	−0.249	0.169

Higher-order statistics features were derived from wavelet transformation including the first-order statistics features and texture features in eight directions (HLL, LLL, HLH, HHL, LLH, LHH, LHL, and HHH).

SRE, short run emphasis; LGLRE, low gray-level run emphasis; HGLRE, high gray-level run emphasis; CS, cluster shade; IMC2, informational measure of correlation 2; LRLGLE, long run low gray-level emphasis; MoCA, Montreal Cognitive Assessment; MMSE, Mini-Mental State Examination; ADL, Activities of Daily Living Scale.

a Significant correlation (p < 0.05).

## Discussion

The early diagnosis of SIVCIND is very important for timely treatment and prognosis improvement. Neuropsychological scale testing, electrophysiology examination, and medical imaging of structural or functional approaches all have various defects that limit their clinical use. Radiomics can automatically extract a large number of high-throughput quantitative features from neuroimaging images using data representation algorithms and provide additional potential information far beyond visual range analysis. Appropriate feature reduction and machine learning methods can obtain the optimal subset of radiographic features and establish a robust and effective diagnostic model. Previously, radiomics analysis of brain MR images was utilized to diagnose mild cognitive impairment (MCI), Alzheimer’s disease (AD), and schizophrenia and differentiate Parkinson’s disease motor subtypes successfully (26). On the other hand, the radiomics of brain MR images could predict the time to progression from MCI to AD (22).

So far, the research on machine learning and radiomics in the diagnosis of SIVCIND has not been reported. In this study, we found that the LR, SVM, and RF models based on the optimal features all yielded excellent performance in terms of SIVCIND diagnosis. Among them, the RF model showed the highest AUC of 0.990 and the highest accuracy of 0.948. These results suggested that the combination of machine learning and radiomics could diagnose SIVCIND accurately. Because of the convenience, objectivity, and non-invasiveness, it has important clinical application value. Previously, graph theory and network-based statistics (NBS) have been employed to analyze the whole-brain mean factional anisotropy matrix, and the accuracy for diagnosing SIVCIND is 0.780 (27). Another study used an SVM-based machine learning strategy to discriminate between different cognitive stages of SIVCI patients with predefined features extracted from diffusion tensor imaging (DTI) and got an accuracy of 0.775–0.800 (28). Wang et al. had used a convolutional neural network (CNN) and T2-weighted sequence to diagnose SIVCIND. The accuracy of the 2D model was 0.540, and that of the 3D model was 0.900 (29). Compared with these previous studies, our study obtained higher AUC and accuracy. The possible reason may be that we analyzed the high-resolution images of the whole cerebral cortex and subcortical nucleus. A large number of high-throughput radiological features can provide more neuropathological features and improve diagnostic accuracy.

In this study, after feature reduction, 13 features from subcortical nuclei of the thalamus, caudate nucleus, nucleus accumbens, putamen, amygdala, and hippocampus were selected as the optimal subset. The radiomics features can reveal hidden changes of brain microstructure that are difficult to be quantitatively recognized by the naked eye. All of the 13 features were intensity features and texture features, which can reflect the heterogeneity of brain tissue images. The heterogeneity of the SIVCIND might be due to the neuron degeneration, lacunar infarcts, and latent lesions originating from subcortical ischemic vascular disease, which often occurs in the area of the basal ganglia and thalamus (30, 31). Among all the 13 features, 11 of them were higher-order statistics features of wavelet conversion. This indicated that higher-order statistics features could disclose the latent changes of brain tissue more explicitly. Wavelet conversion can lessen noise and sharpen the image and does not change the semantic meaning of the radiomics parameters (11). According to the correlation analysis, the radiomics features of the right amygdala, left caudate nucleus, left putamen, and left thalamus were found to be significantly correlated with the neuropsychological scores of the SIVCIND patients. This result suggested that the concealed changes of the above regions may be early biological markers of SIVCIND. The putamen is the area of the brain responsible for emotion and motivation (32, 33). The thalamus and caudate nucleus have been proved to be related to consciousness and cognition (33, 34). The amygdala is associated with exercise and emotion (32, 35).

In conclusion, in this study, we found that the integration of radiomics derived from brain high-resolution T1WI and machine learning could diagnose SIVCIND accurately and automatically. The optimal radiomics features are mostly located in the right amygdala, left caudate nucleus, left putamen, and left thalamus, which might be the new biomarkers of SIVCIND. The high-resolution T1-weighted MR imaging was generated by MP-RAGE sequence, which was easily obtained and widely applied in clinical practice. It can provide images with high spatial resolution and a high signal-to-noise ratio (36). The combination of radiomics derived from brain high-resolution T1WI and automatic machine learning to diagnose SIVCIND is a new technology. This method is independent of the traditional clinical evaluation and can be used as an effective supplement to the traditional neuropsychological scale test.

This study has several limitations. First, the sample size is relatively small due to strict inclusion/exclusion criteria. Second, this research only focused on the structural data of the SIVCIND subjects but did not study the functional data. Third, in the model construction, only MRI-derived radiological features were used. The laboratory measurements and clinical information were not taken into consideration. In future work, different imaging technologies, including CT, PET, and MRI, and clinical and laboratory parameters should be combined to further improve the diagnostic efficiency of SIVCIND.

## Data Availability Statement

The raw data supporting the conclusions of this article will be made available by the authors, without undue reservation.

## Ethics Statement

The studies involving human participants were reviewed and approved by The Second Affiliated Hospital of Chongqing Medical University. The patients/participants provided their written informed consent to participate in this study.

## Author Contributions

BL and SM analyzed the data and wrote the manuscript. JC and HL analyzed the data. YZ, DZ, XD, LK, XW, LT, and HW collected the relevant data. CML and CL put forward the study topic and revised the manuscript. All authors read and approved the final manuscript.

## Funding

This study has received funding from the Chongqing Natural Science Foundation (grant number cstc2020jcyj-msxmX0044), Chongqing Science and Health Joint Medical Research Project of China (grant number 2018ZDXM005), Intelligent Medicine Research Project of Chongqing Medical University (grant number ZHYX202004), Special Project for Technological Innovation and Application Development of Chongqing City (grant number cstc2019jscx-msxmX0104), and Kuanren Talents Program of the Second Affiliated Hospital of Chongqing Medical University.

## Conflict of Interest

Author HL was employed by GE Healthcare (China).

The remaining authors declare that the research was conducted in the absence of any commercial or financial relationships that could be construed as a potential conflict of interest.

## Publisher’s Note

All claims expressed in this article are solely those of the authors and do not necessarily represent those of their affiliated organizations, or those of the publisher, the editors and the reviewers. Any product that may be evaluated in this article, or claim that may be made by its manufacturer, is not guaranteed or endorsed by the publisher.

## Footnotes

1
http://brant.brainnetome.org

## Footnotes

2
https://surfer.nmr.mgh.harvard.edu/
